# Inhibitory effects of astaxanthin on azoxymethane-induced colonic preneoplastic lesions in C57/BL/KsJ-*db/db* mice

**DOI:** 10.1186/s12876-014-0212-z

**Published:** 2014-12-17

**Authors:** Takahiro Kochi, Masahito Shimizu, Takafumi Sumi, Masaya Kubota, Yohei Shirakami, Takuji Tanaka, Hisataka Moriwaki

**Affiliations:** Department of Gastroenterology/Internal Medicine, Gifu University Graduate School of Medicine, 1-1 Yanagido, Gifu, 501-1194 Japan; Department of Tumor Pathology, Gifu University Graduate School of Medicine, Gifu, 501-1194 Japan

**Keywords:** Astaxanthin, Chemoprevention, Preneoplastic lesions, Colon, Obesity, Mice

## Abstract

**Background:**

Obesity and related metabolic abnormalities, including excess oxidative stress and chronic inflammation, are associated with colorectal carcinogenesis. Astaxanthin, a xanthophyll carotenoid found in aquatic animals, is known to possess antioxidant, anti-inflammatory, and antineoplastic properties. The present study examined the effects of astaxanthin on the development of azoxymethane (AOM)-induced colonic premalignant lesions in C57BL/KsJ-*db/db* (*db/db*) obese mice.

**Method:**

Male *db/db* mice were administered 4 weekly subcutaneous injections of AOM (15 mg/kg body weight) from 5 weeks of age and subsequently, from 1 week after the last injection of AOM, were fed a diet containing 200 ppm astaxanthin throughout the experiment (8 weeks).

**Result:**

The development of colonic premalignant lesions, i.e., aberrant crypt foci and β-catenin accumulated crypts, was significantly inhibited in mice treated with astaxanthin than in mice fed the basal diet. Astaxanthin administration markedly reduced urinary levels of 8-OHdG and serum levels of d-ROMs, which are oxidative stress markers, while increasing the expression of mRNA for the antioxidant enzymes *GPx1*, *SOD1*, and *CAT* in the colonic mucosa of AOM-treated *db/db* mice. The expression levels of *IL-1β*, *IL-6*, *F4/80*, *CCL2*, and *CXCL2* mRNA in the colonic mucosa of AOM-treated mice were significantly decreased by astaxanthin. Dietary feeding with astaxanthin also resulted in a reduction in the numbers of NF-κB- and PCNA-positive cells that were increased by AOM exposure, in the colonic epithelium.

**Conclusion:**

These findings suggest that astaxanthin inhibits the development of colonic premalignant lesions in an obesity-related colorectal carcinogenesis model by reducing oxidative stress, attenuating chronic inflammation, and inhibiting NF-κB activation and cell proliferation in the colonic mucosa. Astaxanthin, therefore, may be a potential candidate as a chemoprevention agent against colorectal carcinogenesis in obese individuals.

## Background

Obesity, a growing health concern worldwide, is a result of excess energy intake and insufficient exercise. Obesity is associated with increased risk of diseases with high mortality, such as ischemic heart disease, stroke, and cancer [1]. In particular, the risk of colorectal cancer (CRC), which is the third most common malignancy in men and the second in women, globally [2], is especially higher when combined with obesity [3-5]. The five-year survival for early stage CRC is 80–90% but decreases to 65% for all stages [6]. Therefore, in addition to early detection and treatment, chemoprevention with effective agents is considered extremely important for the comprehensive management of CRC [7,8].

Several pathophysiological mechanisms linking obesity and the development of CRC have been elucidated, including the emergence of insulin resistance, imbalance of adipokines, induction of oxidative stress, and a state of chronic inflammation [3-5]. Obese and diabetic mice are susceptible to chemically induced colon tumorigenesis [9]. Diet-induced obesity significantly promotes colon tumor development in mice [10]. On the other hand, recent studies have demonstrated that certain types of phytochemicals such as curcumin and (−)-epigallocatechin gallate inhibit the development of obesity-related colorectal carcinogenesis in mice by attenuating chronic inflammation [11,12]. Administration of angiotensin-converting enzyme inhibitor also suppresses the early phase of colorectal carcinogenesis in diabetic and hypertensive rats by attenuating inflammation and oxidative stress [13]. These reports suggest that targeting obesity-related metabolic abnormalities including chronic inflammation and oxidative stress using phytochemicals and specific agents is an effective strategy for preventing CRC development in obese individuals [3].

Astaxanthin, a conventional red-colored xanthophyll, is an oxygenated carotenoid derivative occurring naturally in a wide variety of living organisms including microalgae, fungi, salmon, trout, shrimp, and some birds [14,15]. Astaxanthin has been shown to exert numerous pharmacological effects due to its antioxidant, anti-inflammatory, antidiabetic, and antineoplastic properties [8,14,15]. Supplementation with astaxanthin actually decreased oxidative stress and inflammation in a clinical trial [16]. In preclinical animal studies, dietary astaxanthin was found to significantly inhibit chemically induced colorectal [17,18], urinary bladder [19], and oral carcinogenesis [20]. In particular, anti-inflammatory activity is one of the key mechanisms by which astaxanthin prevents colitis-related CRC development [17,18].

C57BL/KsJ-*db/db* (*db/db*) mice, which lack the long form of the leptin receptor, develop hyperphagic obesity and diabetes [21]. Interestingly, the development of colonic premalignant lesions induced by azoxymethane (AOM), a colonic carcinogen widely used to produce preneoplastic and neoplastic colonic lesions that mimic those observed human colon, is significantly enhanced in *db/db* mice [22]. A preclinical animal model using AOM and *db/db* mice [22] has proved useful in investigating specific agents for their ability to prevent inflammation-related colorectal carcinogenesis caused by obesity [11,12,23-25]. In the present study, we investigated the effects of astaxanthin on the development of colonic premalignant lesions, i.e., aberrant crypt foci (ACF) and β-catenin accumulated crypts (BCAC) [26-28], using this obesity-related colorectal carcinogenesis model, with special focus on the reduction of oxidative stress and the attenuation of inflammation.

## Methods

### Animals and chemicals

Four-week-old male *db/db* mice were purchased from Japan SLC, Inc. (Shizuoka, Japan) and were maintained humanely at the Gifu University Life Science Research Center in accordance with the Institutional Animal Care Guidelines. AOM was purchased from Sigma-Aldrich (St. Louis, MO, USA). Astaxanthin from *Haematococcus pluvialiswas* was supplied by Fuji Chemical Industry (Toyama, Japan).

### Experimental procedure

All experimental protocols involving animals were approved by the Animal Research Committee, Gifu University Graduate School of Medicine. Forty male *db/db* mice were divided into the following 4 groups: untreated control (n = 10); 200 ppm astaxanthin alone (n = 10); AOM alone (n = 10); and, AOM and 200 ppm astaxanthin (n = 10). At 5 weeks of age, mice in AOM alone group and AOM + astaxanthin group were injected with AOM (15 mg/kg body weight) subcutaneously once a week for 4 weeks. None treatment group and AOM alone group were fed the basal diet CRF-1 (the formula of Charles River, Oriental Yeast, Tokyo, Japan) throughout the experiment. The composition of the CRF-1 diet was as follows: 8.1 g/100 g water, 22.6 g/100 g protein, 5.6 g/100 g fat, 6.6 g/100 g minerals, 3.3 g/100 g fiber, and 53.8 g/100 g carbohydrates. Astaxanthin alone group and AOM + astaxanthin group were fed the basal diet supplemented with 200 ppm astaxanthin for 8 weeks, starting 1 week after the last injection of AOM. The dosage of astaxanthin was determined according to a previous report [18]. Food intakes in all groups were measured daily, while body weights were recorded once a week during the study. At the termination of the study (17 weeks of age), all mice were killed, and the development of ACF and BCAC was analyzed.

### Identification and quantification of ACF and BCAC

The numbers of ACF and BCAC were determined according to standard procedures [12,23,24,29]. After fixing flat in 10% buffered formalin for 24 hours, we stained the colons with methylene blue (0.5% in distilled water) to count ACF. The number of ACF was recorded along with the number of aberrant crypts (ACs) in each focus. The data are expressed per colon. The distal parts of the colon (1 cm from anus; mean area, 0.7 cm^2^/colon) were then resected and embedded in paraffin, and a total of 20 serial sections (each 4 μm thick) per mouse were cut by an *en face* preparation to identify BCAC intramucosal lesions [12,23,24].

### Immunohistochemical analyses for β-catenin, proliferating cell nuclear antigen, and nuclear factor-κB

Immunohistochemistry for β-catenin was performed using the labeled streptavidin-biotin method (LSAB Kit; DAKO, Glostrup, Denmark) to count the number of BCAC [12,23,24]. The primary antibody for β-catenin (BD Transduction Laboratories, San Jose, CA, USA) was used at a final dilution of 1:1000. Immunohistochemical staining for proliferating cell nuclear antigen (PCNA), which is a G_1_-to-S phase marker, and for phospho-nuclear factor-κB (NF-κB) p65 were run on histological sections to estimate cell proliferative activity and NF-κB activity, respectively, in the colonic crypts [11,23], using the LSAB Kit (DAKO) with primary antibodies, anti-PCNA antibody (a final dilution of 1:100, Santa Cruz Biotechnology, Dallas, TX, USA) and anti-phospho-NF-κB p65 antibody (a final dilution of 1:50, Ser276; Cell Signaling Technology, Danvers, MA, USA). The PCNA-labeling index (%) and positive cell index (%) for phospho-NF-κB p65 were determined based on previous methods [11,23].

### RNA extraction and quantitative real-time reverse transcription-PCR analysis

Total RNA was isolated from scraped colonic mucosa of experimental mice using the RNeasy Mini Kit (QIAGEN, Venlo, Netherlands). The cDNA was synthesized from 0.2 μg of total RNA using the High Capacity cDNA Reverse Transcription Kit (Applied Biosystems, Foster City, CA, USA). A quantitative real-time reverse transcription-PCR (RT-PCR) analysis was performed using a LightCycler Nano (Roche Diagnostics, Indianapolis, IN, USA) with FastStart Essential DNA Green Master (Roche Diagnostics). The PCR cycling conditions were 95°C for 10 min, followed by 45 cycles of 95°C for 10 s, 60°C for 10 s, and 72°C for 15 s. The sequences of specific primers amplifying *tumor necrosis factor (TNF)-α*, *interleukin (IL)-1β*, *IL-6*, *F4/80*, *chemokine (C-C motif) ligand (CCL)2*, *chemokine (C-X-C motif) ligand (CXCL)2*, *glutathione peroxidase (GPx)1*, *superoxide dismutase (SOD)1*, *catalase (CAT)* and *glyceraldehyde-3-phosphate dehydrogenase (GAPDH)* genes were obtained from Primer-BLAST (http://www.ncbi.nlm.nih.gov/tools/primer-blast/; Table 1). The expression levels of *TNF-α*, *IL-1β*, *IL-6*, *F4/80*, *CCL2*, *CXCL2*, *GPx1*, *SDO1,* and *CAT* genes were normalized to the *GAPDH* gene expression levels.

**Table 1:** **Primers sequences**

**Target gene**	**Direction**	**Primer sequence (5’-3”)**
TNF-α	forward	TGTCCCTTTCACTCACTGGC
	reverse	CATCTTTTGGGGGAGTGCCT
IL-1β	forward	GACTTCACCATGGAACCCGT
	reverse	GGAGACTGCCCATTCTCGAC
IL-6	forward	TCCAGTTGCCTTCTTGGGAC
	reverse	AGTCTCCTCTCCGGACTTGT
F4/80	forward	CTGAACATGCAACCTGCCAC
	reverse	TTCACAGGATTCGTCCAGGC
CCL2	forward	GTGCTGACCCCAAGAAGGAA
	reverse	GTGCTGAAGACCTTAGGGCA
CXCL2	forward	GGAAGCCTGGATCGTACCTG
	reverse	TGAAAGCCATCCGACTGCAT
GPx1	forward	GATCCCCAGAGCGTTACTCG
	reverse	GTTGTGGAAACTCACACGCC
CAT	forward	GAAGGACCGTGTTTGGTTGC
	reverse	CCGCTGGCGCTTTTCTTGTT
SOD1	forward	CTTGACCCTGGATTGCAGCC
	reverse	GTTTCGTGAGGAAGCCAGGA
GAPDH	forward	GGACCTCATGGCCTACATGG
	reverse	TAGGGCCTCTCTTGCTCAGT

### Clinical chemistry

Blood samples were collected from the inferior vena cava at sacrifice after 6 hours of fasting for chemical analyses. The serum concentrations of insulin (Shibayagi, Gunma, Japan), glucose (BioVision Research Products, Mountain View, CA, USA), adiponectin (R&D Systems, Minneapolis, MN, USA), leptin (R&D Systems), triglyceride (Wako, Osaka, Japan), and TNF-α (R&D Systems) were determined using an enzyme immunoassay, according to the manufacturer’s protocol.

### Oxidative stress analysis

Urine 8-hydroxy-2’-deoxyguanosine (8-OHdG) levels were measured using an enzyme-linked immunosorbent assay kit (NIKKEN SEIL, Shizuoka, Japan). Serum levels of hydroperoxide, a marker for oxidative stress, were determined using the derivatives of reactive oxygen metabolites (d-ROMs) test (FREE Carpe Diem, Diacron International s.r.l., Grosseto, Italy) [30].

### Statistical analyses

The measures are presented as mean ± SD and were statistically analyzed using the GraphPad InStat software program, Version 3.05 (GraphPad Software, San Diego, CA, USA) for Macintosh. One-way analysis of variance (ANOVA) was used to compare groups. If the ANOVA analysis indicated significant differences, the Tukey–Kramer multiple comparisons test was performed to compare the mean values among the groups. The differences were considered significant when the two-sided *P* value was less than 0.05.

## Results

### General observations

During the experiment, dietary feeding with astaxanthin did not cause any clinical symptoms. As listed in Table 2, the mean body weight of the AOM alone group was lower than that of AOM-untreated control group (group 1, *P* < 0.05) at the termination of the experiment. This might be due to the toxicity of AOM, as observed in our previous studies [12,23,24]. The mean relative liver weight (g/100 g body weight) of AOM alone group was also significantly lower than that of none treatment group (*P* < 0.05). No significant differences were observed in the mean relative weights of adipose tissue among the groups. Histopathological examination of the liver, kidney, and spleen confirmed the absence of toxicity of dietary astaxanthin (data not shown).

**Table 2:** **Body, liver and adipose weights of the experimental mice**

**Group no.**	**Treatment**	**No. of mice**	**Body weight (g)** ^**a**^	**Relative organ weight (g/100 g body weight)** ^**a**^	
				**Liver**	**adipose** ^**b**^
1	None	10	51.2 ± 3.2	6.7 ± 0.9	5.9 ± 0.5
2	Astaxanthin	10	50.3 ± 3.0	6.1 ± 0.8	5.5 ± 0.8
3	AOM alone	10	46.7 ± 4.0^c^	5.6 ± 0.7^c^	5.6 ± 0.8
4	AOM + astaxanthin	10	48.6 ± 3.4	6.0 ± 0.6	5.1 ± 0.7

^a^Mean ± SD.

^b^White adipose tissue of the periorchis and retroperitoneum.

^c^Significantly different from group 1 by Tukey-Kramer Multiple Comparison Test (*P* < 0.05).

### Effects of astaxanthin on AOM-induced ACF and BCAC formation in experimental mice

**Figure 1:**
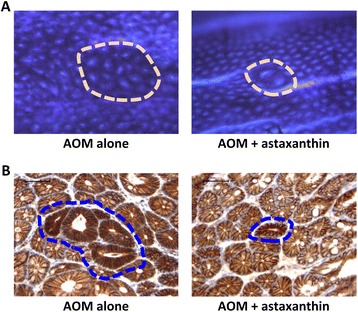
**Effects of astaxanthin on AOM-induced ACF and BCAC formation in experimental mice. (A)** Representative morphology of ACF (arrows) induced by AOM, revealed by methylene blue staining in an AOM alone-injected mouse (left panel) and AOM + astaxanthin-treated mouse (right panel). **(B)** β-catenin immunohistochemistry of BCAC (broken line) that developed in an AOM alone-injected mouse (left panel) and AOM + astaxanthin-treated mouse (right panel).

ACF (Figure 1A) developed only in the colons of mice that received AOM. The total mean numbers of ACF/colon, aberrant crypts/colon, and large ACFs/colon were significantly reduced by astaxanthin administration (Table 3; *P* < 0.05). Moreover, the number of BCAC (Figure 1B), which also developed only in the colons of AOM-treated mice, markedly decreased when these mice were fed astaxanthin-containing diets (Table 3; *P* < 0.05), indicating that two types of precursor lesions for CRC were suppressed by astaxanthin administration. These findings suggest that astaxanthin prevented the early phase of obesity-related colorectal carcinogenesis.

**Table 3:** **Effects of astaxanthin on AOM-induced and BCAC formations in** ***db/db*** **mice**

**Group no.**	**Treatment**	**No. of mice**	**Total no. of ACF/colon** ^**a**^	**Total no. of ACF/colonaCrypt multiplicity (Average no. of crypts/foci)** ^**a**^	**Total no. of large ACFs** ^**b**^ **/colon** ^**a**^	**Total no. of BCACs/cm2** ^**a**^
1	None	10	0	0	0	0
2	Astaxanthin	10	0	0	0	0
3	AOM alone	10	11.9 ± 7.2	27.4 ± 12.5	1.4 ± 1.5	5.9 ± 2.6
4	AOM + astaxanthin	10	4.3 ± 2.7^c^	13.5 ± 5.7^c^	0.3 ± 0.6^c^	1.0 ± 1.0^c^

^a^Mean ± SD.

^b^“Large ACFs” are ACFs with four or more aberrant crypts.

^c^ Significantly different from group 3 by Tukey-Kramer Multiple Comparison Test (*P* < 0.05).

### Effects of astaxanthin on systemic oxidative stress and expression levels of *GPx1*, *SOD1*, and *CAT* mRNA in the colonic mucosa of experimental mice

**Figure 2:**
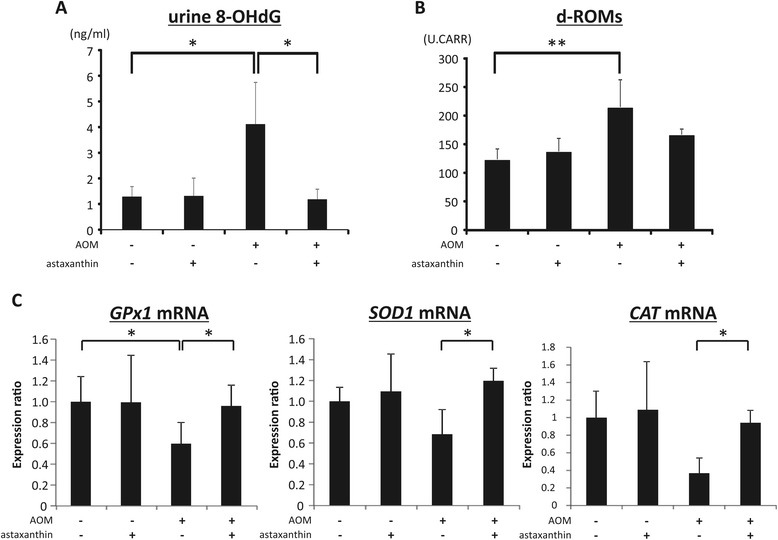
**Effects of astaxanthin on levels of urinary 8-OHdG, serum levels of d-ROMs, and colonic expression levels of** ***GPx1*** **,** ***SOD1*** **, and** ***CAT*** **mRNA in experimental mice. (A)** At the time of killing, urine samples were collected from experimental mice, and levels of urinary 8-OHdG were measured by enzyme-linked immunosorbent assay. **(B)** Hydroperoxide levels in the serum were determined by the d-ROM test. **(C)** Total RNA was isolated from the colonic mucosa of experimental mice, and the expression levels of *GPx1*, *SOD1*, and *CAT* mRNA were examined by quantitative real-time RT-PCR using specific primers. Values are expressed as mean ± SD (triple assays). *P < 0.05, **P < 0.01

Oxidative stress is implicated in obesity-related colorectal tumorigenesis [4]. Therefore, we determined the levels of oxidative stress and antioxidant biomarkers in the experimental mice. AOM injection significantly increased the levels of urinary 8-OHdG (Figure 2A; *P* < 0.05), which reflects DNA damage induced by oxidative stress, and serum d-ROMs (Figure 2B; *P* < 0.01), marker for hydroperoxide levels of serum, as compared to those observed in the control group. However, the level of 8-OHdG significantly decreased with astaxanthin administration (Figure 2A; *P* < 0.05). As shown in Figure 2C, the expression of *GPx1* mRNA, which encodes an antioxidant enzyme, was reduced by AOM injection (*P* < 0.05), but astaxanthin significantly increased the expression of *GPx1*, as well as of *SOD1* and *CAT*, both of which encode antioxidant enzymes, in the colonic mucosa of *db/db* mice treated with AOM (*P* < 0.05).

### Effects of astaxanthin on serum levels of TNF-α and expression levels of *TNF-α*, *IL-1β*, *IL-6*, *F4/80*, *CCL2*, and *CXCL2* mRNA in the colonic mucosa of experimental mice

**Figure 3:**
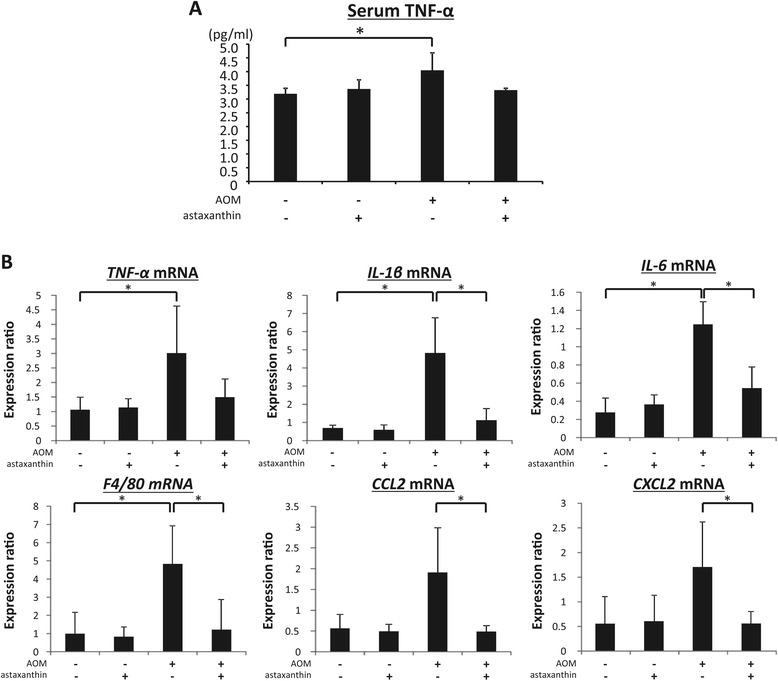
**Effects of astaxanthin on serum levels of TNF-α and colonic expression levels of** ***TNF-α*** **,** ***IL-1β*** **,** ***IL-6*** **,** ***F4/80*** **,** ***CCL2*** **, and** ***CXCL2*** **mRNA in experimental mice. (A)** The serum concentration of TNF-α was measured by enzyme immunoassay. **(B)** Total RNA was isolated from the colonic mucosa of experimental mice, and the expression levels of *TNF-α*, *IL-1β*, *IL-6*, *F4/80*, *CCL2*, and *CXCL2* mRNA were examined by quantitative real-time RT-PCR using specific primers. Values are expressed as mean ± SD (triple assays). *P < 0.05

Chronic inflammation plays a critical role in the pathogenesis of obesity and CRC development [4,31]. Therefore, the effect of astaxanthin on the levels of inflammatory mediators including TNF-α, IL-1β, IL-6, F4/80, CCL2, and CXCL2 in experimental mice was examined. Serum levels of TNF-α were significantly higher in the AOM-injected mice (Figure 3A; *P* < 0.05), but not in the astaxanthin-treated mice, than those in the control mice. As shown in Figure 3B, there was a marked increase in the expression levels of *TNF-α*, *IL-1β*, *IL-6*, and *F4/80* (*P* < 0.05 compared to the untreated control group) in the colonic mucosa of group treated with AOM alone. However, astaxanthin administration significantly decreased the expression of *IL-1β*, *IL-6*, and *F4/80* mRNA in the colonic mucosa of the AOM-treated *db/db* mice (*P* < 0.05). The expression of *CCL2* and *CXCL2*, which are associated with colorectal carcinogenesis [32-34], in the colonic mucosa of the AOM-treated *db/db* mice was also significantly decreased by astaxanthin treatment (*P* < 0.05).

### Effects of astaxanthin on NF-κB activity and cell proliferative activity in the colonic mucosa of experimental mice

**Figure 4:**
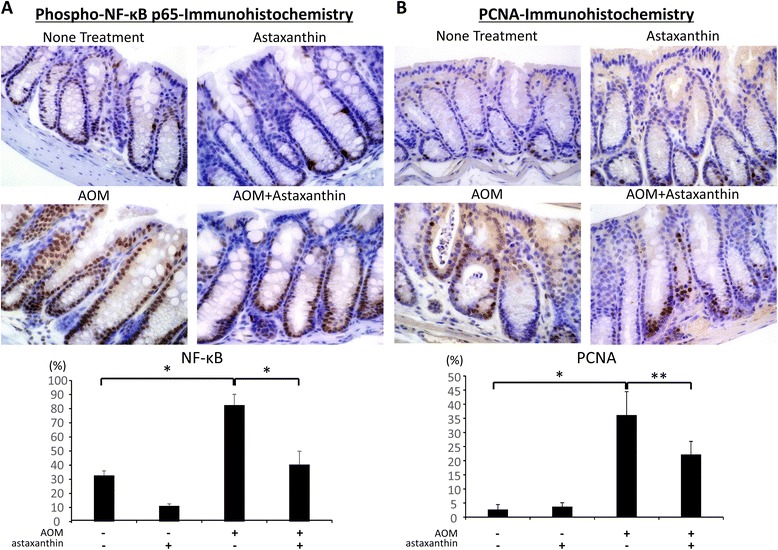
**Effects of astaxanthin on the NF-κB activity and cell proliferative activity in the colonic mucosa of experimental mice.** Sections of the colon were stained with **(A)** anti-phospho-NF-κB p65 or **(B)** anti-PCNA antibodies, respectively. Representative photographs from each group are shown in the upper panels. The positive cell indices, which were determined by counting **(A)** phospho-NF-κB p65-positive cells or **(B)** PCNA-positive cells in the colonic crypts, are shown in the lower panels. Original magnification, 200×. Bars, SD of triplicate assays. *P < 0.001, **P < 0.01

NF-κB activation is critically involved in the progression of inflammation and the activation of cell proliferation in colonic mucosa [35]. Therefore, the effects of astaxanthin on NF-κB activity and cell proliferative activity were examined in the colonic mucosa of experimental mice. The indices of phospho-NF-κB p65-positive cells, which were increased by AOM injection in the colonic epithelium, were significantly reduced by astaxanthin treatment (Figure 4A; *P* < 0.001 for each comparison). As shown in Figure 4B, astaxanthin treatment also significantly decreased the PCNA-labeling indices of non-lesional crypts (*P* < 0.01), which had been increased by AOM injection (*P* < 0.001). These findings indicate that astaxanthin significantly inhibits NF-κB activity and cell proliferation in the colonic mucosa of AOM-treated *db/db* mice.

### Effects of astaxanthin on serum parameters in experimental mice

Diabetes, dyslipidemia, and adipokine imbalance, which are complications often associated with obesity, are involved in colorectal tumorigenesis [3-5]. Therefore, serum parameters associated with these metabolic disorders were assessed. AOM exposure did not affect either serum levels of adiponectin, triglycerides, glucose, and insulin or the value of QUICKI, a useful index of insulin sensitivity [36], in experimental mice (Table 4). The serum level of leptin was significantly elevated by AOM irrespective of astaxanthin administration (*P* < 0.05). None of these metabolic parameters had been altered by astaxanthin administration at the end of this study.

**Table 4:** **Serum parameters in the experimental mice**

**Group no. treatment**	**1 None** ^**a**^	**2 Astaxanthin** ^**a**^	**3 AOM alone** ^**a**^	**4 AOM + astaxanthin** ^**a**^
Glucose (mg/dl)	110.8 ± 18.4	110.2 ± 18.3	97.6 ± 24.6	83.6 ± 25.1
Insulin (nM/ml)	131.8 ± 53.3	120.7 ± 35.3	203.1 ± 51.9	174.9 ± 47.3
Quicki	0.249 ± 0.005	0.253 ± 0.007	0.238 ± 0.007	0.238 ± 0.011
Adiponectin (ng/ml)	52.2 ± 2.0	53.3 ± 1.1	57.1 ± 13.8	57.5 ± 3.2
Leptin (ng/ml)	5.3 ± 0.3	4.6 ± 0.5	9.9 ± 0.3^b^	8.3 ± 0.5^c^
Triglyceride (mg/dl)	91.6 ± 32.7	91.6 ± 35.4	82.7 ± 24.6	90.2 ± 50.1

^a^Mean ± SD.

^b^Significantly different from group 1 by Tukey-Kramer Multiple Comaprison Test (P < 0.05).

^c^Significantly different from group 3 by Tukey-Kramer Multiple Comaprison Test (P < 0.05).

## Discussion

Obesity, which is one of the most serious healthcare problems worldwide, is a significant risk factor for the development of CRC [4,5]. Oxidative stress and chronic inflammation are key mechanisms linking obesity and colorectal carcinogenesis [37,38]. In particular, increased adipose tissue creates an oxidative environment that can upregulate the expression of various pro-inflammatory cytokines including TNF-α and IL-6, and this is critically associated with CRC development because these cytokines stimulate tumor growth and progression [31,39]. The results of the present study show that astaxanthin exerts preventive effects on the development of the AOM-induced colonic premalignant lesions ACF [26,27] and BCAC [28] in *db/db* obese mice, mainly through the reduction of oxidative stress and attenuation of inflammation.

Saturated fatty acids from obesity-induced lipolysis are capable of activating macrophages and thereby activating NF-κB signaling, which in turn leads to transcriptional activation of genes encoding pro-inflammatory factors including IL-1β and IL-6 [39]. IL-1β plays a key role in obesity-induced inflammation [39] and inflammation-related carcinogenesis by modulating the gene expression involved in proliferation, survival, and angiogenesis [40]. The expression of CCL2, which is associated with infiltration and migration of tumor-related macrophages, has been demonstrated in several tumor tissues including CRC [32,33]. In addition, CXCL2, an inflammatory cytokine, has been shown to participate in the early phase of colorectal carcinogenesis [34]. In the present study, the expression levels of IL-1β, IL-6, F4/80, CCL2, and CXCL2 mRNA were decreased by astaxanthin in the experimental mice. These findings indicate that overexpression of these inflammatory mediators, which connect obesity and carcinogenesis, may be one of the critical targets of astaxanthin in preventing the development of obesity-related CRC. These findings are also consistent with those from a previous study showing that feeding with astaxanthin significantly suppressed colitis and colitis-related colorectal carcinogenesis by inhibiting NF-κB activation, decreasing the expression of IL-1β and IL-6, and suppressing cell proliferation in mice [18]. In particular, NF-κB signaling pathway is regarded as one of the key targets of astaxanthin to exert chemopreventive effects [41].

Obesity and chronic inflammation are often accompanied with increased generation of reactive oxygen species (ROS), which are derivatives of molecular oxygen such as superoxide and hydrogen peroxide. These derivatives can induce mutagenic changes and may damage DNA repair proteins, resulting in cancer development [40,42]. Cancer cells are also known to cause oxidative stress by generating ROS and modulating antioxidant enzymes [43]. In the present study, oxidative stress markers, including urinary levels of 8-OHdG and serum levels of d-ROMs, were significantly suppressed, while the expression levels of *GPx1*, *SOD1*, and *CAT* mRNA, which encode antioxidant enzymes, in the colonic mucosa were increased by astaxanthin intake in AOM-injected *db/db* mice. These results strongly indicate that attenuation of oxidative stress and recuperation from high oxidation state via antioxidative effects are critical mechanisms by which astaxanthin suppressed the occurrence of premalignant lesions, ACF and BCAC, in obese mice. It should be mentioned that astaxanthin has even been called a super-antioxidant because it is a superior antioxidant and scavenger of free radicals as compared with other carotenoids such as β-carotene [44].

Epidemiologically, it is still unclear whether the intake of carotenoids such as astaxanthin is associated with a reduced risk of CRC development. The results of randomized trials using β-carotene supplementation provided no evidence to support an effect of carotenoids on CRC chemoprevention [45,46]. Rather, intervention trials using high-dose β-carotene supplements showed an increase in the incidence of lung cancer in high-risk patients, like smokers and/or workers exposed to asbestos [47,48]. On the other hand, astaxanthin has been demonstrated to be safe in several human clinical trials [16,49,50]. Moreover, astaxanthin supplementation has positive effects on lipid profiles and oxidative stress in overweight and obese subjects, at least in part by activating the antioxidant defense system [49,50]. Taken together, these observations suggest that obese individuals, who are at high-risk of developing CRC and colorectal adenomas [5], may be appropriate subjects for interventional trials using astaxanthin for the prevention of colorectal tumorigenesis.

Previous reports using metabolic syndrome animal models have shown that astaxanthin reduces insulin resistance, recovers insulin sensitivity, and increases serum levels of adiponectin [51,52]. A double-blind randomized controlled trial also reported that astaxanthin consumption significantly increases blood adiponectin levels [53]. Furthermore, targeting insulin resistance and adipokine imbalance are suggested to be effective methods of preventing obesity-related colorectal tumorigenesis [3]. Therefore, we initially expected that astaxanthin would inhibit the development of ACF and BCAC in the AOM-treated *db/db* mice by ameliorating insulin resistance and improving adipokine imbalance. However, abnormalities in serum levels of glucose, insulin, adiponectin, and leptin were not improved by astaxanthin administration in this study. We suggest that this was likely due to the duration of the experiment (8 weeks) and the particular animal model studied, because previous studies demonstrating effects of astaxanthin on insulin sensitivity were long-term studies (22 weeks) [51] and the animals were not genetically obese [51,52]. Our results were consistent with another study investigating the effect of astaxanthin on the apoptosis of retinal ganglion cells using *db/db* mice, in which insulin resistance was not improved by astaxanthin, but apoptosis of retinal ganglion cells was attenuated via the suppression of oxidative stress [54]. Future long-term studies should be conducted to confirm that astaxanthin inhibits the early phase of obesity-related colon tumorigenesis by improving insulin resistance and the imbalance of adipokines in several animal models. In our experimental model, astaxanthin suppressed the development of obesity-related colorectal tumorigenesis by targeting oxidative stress, inflammation, and cell proliferation.

## Conclusion

In summary, the results from this study showed that reduction of oxidative stress and attenuation of inflammation in the colonic mucosa are crucial mechanisms by which astaxanthin acts to prevent the early phase of obesity-related colorectal carcinogenesis. Since the risk of CRC increases with obesity and obesity-related metabolic abnormalities, which are impending health crises worldwide, targeting obesity-related metabolic abnormalities, including chronic inflammation and induction of oxidative stress, may be an attractive and effective strategy for preventing the development of CRC in obese individuals. Astaxanthin seems to be a potentially effective and viable candidate for this purpose because this agent attenuates chronic inflammation while reducing oxidative stress without causing side effects.

## Abbreviations

8-OHdG: 8-hydroxy-2ʹ-deoxyguanosine
ACF: Aberrant crypt foci
AOM: Azoxymethane
ANOVA: Analysis of variance
BCAC: β-catenin accumulated crypts
CAT: Catalase
CCL: Chemokine (C-C motif) ligand
CRC: colorectal cancer
CXCL: Chemokine (C-X-C motif) ligand
*db/db*: C57BL/KsJ-*db/db*
d-ROMs: Derivatives of reactive oxygen metabolites
GAPDH: Glyceraldehyde 3-phosphate dehydrogenase
GPx: Glutathione peroxidase
IL: Interleukin
NF-κB: Nuclear factor-κB
PCNA: Proliferating cell nuclear antigen
RT-PCR: Reverse transcription PCR
SOD: Superoxide dismutase
TNF: Tumor necrosis factor

## References

[1] Prospective Studies C, Whitlock G, Lewington S, Sherliker P, Clarke R, Emberson J, Halsey J, Qizilbash N, Collins R, Peto R (2009). Body-mass index and cause-specific mortality in 900 000 adults: collaborative analyses of 57 prospective studies. Lancet.

[2] Ferlay J, Shin HR, Bray F, Forman D, Mathers C, Parkin DM (2010). Estimates of worldwide burden of cancer in 2008: GLOBOCAN 2008. Int J Cancer.

[3] Shimizu M, Kubota M, Tanaka T, Moriwaki H (2012). Nutraceutical approach for preventing obesity-related colorectal and liver carcinogenesis. Int J Mol Sci.

[4] Ishino K, Mutoh M, Totsuka Y, Nakagama H (2013). Metabolic syndrome: a novel high-risk state for colorectal cancer. Cancer Lett.

[5] Pais R, Silaghi H, Silaghi AC, Rusu ML, Dumitrascu DL (2009). Metabolic syndrome and risk of subsequent colorectal cancer. World J Gastroenterol.

[6] Siegel R, Ward E, Brawley O, Jemal A (2011). Cancer statistics, 2011: the impact of eliminating socioeconomic and racial disparities on premature cancer deaths. CA Cancer J Clin.

[7] Umar A, Dunn BK, Greenwald P (2012). Future directions in cancer prevention. Nat Rev Cancer.

[8] Tanaka T, Shnimizu M, Moriwaki H (2012). Cancer chemoprevention by carotenoids. Molecules.

[9] Hata K, Kubota M, Shimizu M, Moriwaki H, Kuno T, Tanaka T, Hara A, Hirose Y (2012). Monosodium glutamate-induced diabetic mice are susceptible to azoxymethane-induced colon tumorigenesis. Carcinogenesis.

[10] Tuominen I, Al-Rabadi L, Stavrakis D, Karagiannides I, Pothoulakis C, Bugni JM (2013). Diet-induced obesity promotes colon tumor development in azoxymethane-treated mice. PLoS One.

[11] Kubota M, Shimizu M, Sakai H, Yasuda Y, Terakura D, Baba A, Ohno T, Tsurumi H, Tanaka T, Moriwaki H (2012). Preventive effects of curcumin on the development of azoxymethane-induced colonic preneoplastic lesions in male C57BL/KsJ-db/db obese mice. Nutr Cancer.

[12] Shimizu M, Shirakami Y, Sakai H, Adachi S, Hata K, Hirose Y, Tsurumi H, Tanaka T, Moriwaki H (2008). (−)-Epigallocatechin gallate suppresses azoxymethane-induced colonic premalignant lesions in male C57BL/KsJ-db/db mice. Cancer Prev Res.

[13] Kochi T, Shimizu M, Ohno T, Baba A, Sumi T, Kubota M, Shirakami Y, Tsurumi H, Tanaka T, Moriwaki H (2014). Preventive effects of the angiotensin-converting enzyme inhibitor, captopril, on the development of azoxymethane-induced colonic preneoplastic lesions in diabetic and hypertensive rats. Oncol Lett.

[14] Yuan JP, Peng J, Yin K, Wang JH (2011). Potential health-promoting effects of astaxanthin: a high-value carotenoid mostly from microalgae. Mol Nutr Food Res.

[15] Kidd P (2011). Astaxanthin, cell membrane nutrient with diverse clinical benefits and anti-aging potential. Altern Med Rev.

[16] Park JS, Chyun JH, Kim YK, Line LL, Chew BP (2010). Astaxanthin decreased oxidative stress and inflammation and enhanced immune response in humans. Nutr Metab.

[17] Nagendraprabhu P, Sudhandiran G (2011). Astaxanthin inhibits tumor invasion by decreasing extracellular matrix production and induces apoptosis in experimental rat colon carcinogenesis by modulating the expressions of ERK-2, NFkB and COX-2. Investig New Drugs.

[18] Yasui Y, Hosokawa M, Mikami N, Miyashita K, Tanaka T (2011). Dietary astaxanthin inhibits colitis and colitis-associated colon carcinogenesis in mice via modulation of the inflammatory cytokines. Chem Biol Interact.

[19] Tanaka T, Morishita Y, Suzui M, Kojima T, Okumura A, Mori H (1994). Chemoprevention of mouse urinary bladder carcinogenesis by the naturally occurring carotenoid astaxanthin. Carcinogenesis.

[20] Tanaka T, Makita H, Ohnishi M, Mori H, Satoh K, Hara A (1995). Chemoprevention of rat oral carcinogenesis by naturally occurring xanthophylls, astaxanthin and canthaxanthin. Cancer Res.

[21] Fellmann L, Nascimento AR, Tibirica E, Bousquet P (2013). Murine models for pharmacological studies of the metabolic syndrome. Pharmacol Ther.

[22] Hirose Y, Hata K, Kuno T, Yoshida K, Sakata K, Yamada Y, Tanaka T, Reddy BS, Mori H (2004). Enhancement of development of azoxymethane-induced colonic premalignant lesions in C57BL/KsJ-db/db mice. Carcinogenesis.

[23] Shimizu M, Shirakami Y, Iwasa J, Shiraki M, Yasuda Y, Hata K, Hirose Y, Tsurumi H, Tanaka T, Moriwaki H (2009). Supplementation with branched-chain amino acids inhibits azoxymethane-induced colonic preneoplastic lesions in male C57BL/KsJ-db/db mice. Clin Cancer Res.

[24] Yasuda Y, Shimizu M, Shirakami Y, Sakai H, Kubota M, Hata K, Hirose Y, Tsurumi H, Tanaka T, Moriwaki H (2010). Pitavastatin inhibits azoxymethane-induced colonic preneoplastic lesions in C57BL/KsJ-db/db obese mice. Cancer Sci.

[25] Kubota M, Shimizu M, Sakai H, Yasuda Y, Ohno T, Kochi T, Tsurumi H, Tanaka T, Moriwaki H (2011). Renin-angiotensin system inhibitors suppress azoxymethane-induced colonic preneoplastic lesions in C57BL/KsJ-db/db obese mice. Biochem Biophys Res Commun.

[26] Bird RP, Good CK (2000). The significance of aberrant crypt foci in understanding the pathogenesis of colon cancer. Toxicol Lett.

[27] Raju J (2008). Azoxymethane-induced rat aberrant crypt foci: relevance in studying chemoprevention of colon cancer. World J Gastroenterol.

[28] Yamada Y, Mori H (2003). Pre-cancerous lesions for colorectal cancers in rodents: a new concept. Carcinogenesis.

[29] Bird RP (1987). Observation and quantification of aberrant crypts in the murine colon treated with a colon carcinogen: preliminary findings. Cancer Lett.

[30] Kochi T, Shimizu M, Terakura D, Baba A, Ohno T, Kubota M, Shirakami Y, Tsurumi H, Tanaka T, Moriwaki H (2014). Non-alcoholic steatohepatitis and preneoplastic lesions develop in the liver of obese and hypertensive rats: suppressing effects of EGCG on the development of liver lesions. Cancer Lett.

[31] Donohoe CL, Pidgeon GP, Lysaght J, Reynolds JV (2010). Obesity and gastrointestinal cancer. Br J Surg.

[32] Bailey C, Negus R, Morris A, Ziprin P, Goldin R, Allavena P, Peck D, Darzi A (2007). Chemokine expression is associated with the accumulation of tumour associated macrophages (TAMs) and progression in human colorectal cancer. Clin Exp Metastasis.

[33] Erreni M, Mantovani A, Allavena P (2011). Tumor-associated Macrophages (TAM) and Inflammation in Colorectal Cancer. Cancer Microenviron.

[34] McLean MH, Murray GI, Stewart KN, Norrie G, Mayer C, Hold GL, Thomson J, Fyfe N, Hope M, Mowat NA, Drew JE, El-Omar EM (2011). The inflammatory microenvironment in colorectal neoplasia. PLoS One.

[35] Karin M, Greten FR (2005). NF-kappaB: linking inflammation and immunity to cancer development and progression. Nat Rev Immunol.

[36] Chen H, Sullivan G, Yue LQ, Katz A, Quon MJ (2003). QUICKI is a useful index of insulin sensitivity in subjects with hypertension. Am J Physiol Endocrinol Metab.

[37] Giovannucci E, Michaud D (2007). The role of obesity and related metabolic disturbances in cancers of the colon, prostate, and pancreas. Gastroenterology.

[38] Gunter MJ, Leitzmann MF (2006). Obesity and colorectal cancer: epidemiology, mechanisms and candidate genes. J Nutr Biochem.

[39] Howe LR, Subbaramaiah K, Hudis CA, Dannenberg AJ (2013). Molecular pathways: adipose inflammation as a mediator of obesity-associated cancer. Clin Cancer Res.

[40] Sethi G, Shanmugam MK, Ramachandran L, Kumar AP, Tergaonkar V (2012). Multifaceted link between cancer and inflammation. Biosci Rep.

[41] Kavitha K, Kowshik J, Kishore TK, Baba AB, Nagini S (2013). Astaxanthin inhibits NF-kappaB and Wnt/beta-catenin signaling pathways via inactivation of Erk/MAPK and PI3K/Akt to induce intrinsic apoptosis in a hamster model of oral cancer. Biochim Biophys Acta.

[42] Schetter AJ, Heegaard NH, Harris CC (2010). Inflammation and cancer: interweaving microRNA, free radical, cytokine and p53 pathways. Carcinogenesis.

[43] Mantovani G, Maccio A, Madeddu C, Mura L, Gramignano G, Lusso MR, Massa E, Mocci M, Serpe R (2003). Antioxidant agents are effective in inducing lymphocyte progression through cell cycle in advanced cancer patients: assessment of the most important laboratory indexes of cachexia and oxidative stress. J Mol Med.

[44] Pashkow FJ, Watumull DG, Campbell CL (2008). Astaxanthin: a novel potential treatment for oxidative stress and inflammation in cardiovascular disease. Am J Cardiol.

[45] Cook NR, Le IM, Manson JE, Buring JE, Hennekens CH (2000). Effects of beta-carotene supplementation on cancer incidence by baseline characteristics in the Physicians’ Health Study (United States). Cancer Causes Control.

[46] Albanes D, Malila N, Taylor PR, Huttunen JK, Virtamo J, Edwards BK, Rautalahti M, Hartman AM, Barrett MJ, Pietinen P, Hartman TJ, Sipponen P, Lewin K, Teerenhovi L, Hietanen P, Tangrea JA, Virtanen M, Heinonen OP (2000). Effects of supplemental alpha-tocopherol and beta-carotene on colorectal cancer: results from a controlled trial (Finland). Cancer Causes Control.

[47] Albanes D, Heinonen OP, Taylor PR, Virtamo J, Edwards BK, Rautalahti M, Hartman AM, Palmgren J, Freedman LS, Haapakoski J, Barrett MJ, Pietinen P, Malila N, Tala E, Liippo K, Salomaa ER, Tangrea JA, Teppo L, Askin FB, Taskinen E, Erozan Y, Greenwald P, Huttunen JK (1996). Alpha-Tocopherol and beta-carotene supplements and lung cancer incidence in the alpha-tocopherol, beta-carotene cancer prevention study: effects of base-line characteristics and study compliance. J Natl Cancer Inst.

[48] Omenn GS, Goodman GE, Thornquist MD, Balmes J, Cullen MR, Glass A, Keogh JP, Meyskens FL, Valanis B, Williams JH, Barnhart S, Cherniack MG, Brodkin CA, Hammar S (1996). Risk factors for lung cancer and for intervention effects in CARET, the Beta-Carotene and Retinol Efficacy Trial. J Natl Cancer Inst.

[49] Choi HD, Kim JH, Chang MJ, Kyu-Youn Y, Shin WG (2011). Effects of astaxanthin on oxidative stress in overweight and obese adults. Phytother Res.

[50] Choi HD, Youn YK, Shin WG (2011). Positive effects of astaxanthin on lipid profiles and oxidative stress in overweight subjects. Plant Foods Hum Nutr.

[51] Hussein G, Nakagawa T, Goto H, Shimada Y, Matsumoto K, Sankawa U, Watanabe H (2007). Astaxanthin ameliorates features of metabolic syndrome in SHR/NDmcr-cp. Life Sci.

[52] Arunkumar E, Bhuvaneswari S, Anuradha CV (2012). An intervention study in obese mice with astaxanthin, a marine carotenoid–effects on insulin signaling and pro-inflammatory cytokines. Food Funct.

[53] Yoshida H, Yanai H, Ito K, Tomono Y, Koikeda T, Tsukahara H, Tada N (2010). Administration of natural astaxanthin increases serum HDL-cholesterol and adiponectin in subjects with mild hyperlipidemia. Atherosclerosis.

[54] Dong LY, Jin J, Lu G, Kang XL (2013). Astaxanthin attenuates the apoptosis of retinal ganglion cells in db/db mice by inhibition of oxidative stress. Mar Drugs.

